# Structural Analysis of Sortase A Inhibitors

**DOI:** 10.3390/molecules21111591

**Published:** 2016-11-22

**Authors:** Georgiana Nitulescu, Anca Zanfirescu, Octavian Tudorel Olaru, Isabela Madalina Nicorescu, George Mihai Nitulescu, Denisa Margina

**Affiliations:** Faculty of Pharmacy, “Carol Davila” University of Medicine and Pharmacy, Traian Vuia 6, Bucharest 020956, Romania; georgiana.nitulescu88@gmail.com (G.N.); zanfirescuanca@yahoo.com (A.Z.); octav_olaru2002@yahoo.com (O.T.O.); isabela_nicorescu@yahoo.com (I.M.N.); denisa.margina@gmail.com (D.M.)

**Keywords:** cysteine transpeptidases, anti-virulence drugs, Gram-positive bacteria, classification method, rotatable bonds, hydrogen bond acceptors, Bemis-Murcko scaffolding

## Abstract

Bacterial sortases are cysteine transpeptidases that regulate the covalent linkage of several surface protein virulence factors in Gram-positive bacteria. Virulence factors play significant roles in adhesion, invasion of host tissues, biofilm formation and immune evasion, mediating the bacterial pathogenesis and infectivity. Therefore, sortases are emerging as important targets for the design of new anti-infective agents. We employed a computational study, based on structure derived descriptors and molecular fingerprints, in order to develop simple classification methods which could allow predicting low active or high active SrtA inhibitors. Our results indicate that a highly active SrtA inhibitor has a molecular weight ranging between 180 and 600, contains one up to four nitrogen atoms, up to three oxygen atoms and under 18 hydrogen atoms. Also the hydrogen acceptor number and the molecular flexibility, as assessed by the number of rotatable bounds, have emerged as the most relevant descriptors for SrtA affinity. The Bemis-Murcko scaffolding revealed favoured scaffolds as containing at least two ring structures bonded directly or merged in a condensed cycle. This data represent a valuable tool for identifying new potent SrtA inhibitors, potential anti-virulence agents targeted against Gram-positive bacteria, including multiresistant strains.

## 1. Introduction

Bacteria possess a very good ability to develop efficient drug resistance mechanisms, thus limiting the available therapeutic options against infectious diseases [1]. Disruption of the bacterial viability by bactericidal compounds or inhibition of the growth cycle by bacteriostatic agents leads to a rapid selection of drug-resistant or even multidrug-resistant populations [2]. In this context, new methods to fight bacterial infections are needed. Such are those based on the inhibition of virulence factors rather than on bacterial growth. This strategy applies lower evolutionary pressure as most virulence traits are not essential for bacterial survival [3]. Several virulence pathways are under investigation for the development of anti-virulence therapies, including adhesion, secretion and toxin production [4].

The sortases emerged as important targets new agents designed to block the pathogenesis of Gram-positive bacteria, like staphylococci, streptococci, or enterococci. Bacterial sortases are cysteine transpeptidases regulating the secretion and anchoring of many cell wall proteins with significant roles in bacterial adhesion and invasion of host tissues, biofilm formation, and immune evasion by inhibition of opsonization and phagocytosis [5]. Based on the localization of the catalytic domain there are two types of sortases: type I harbouring an N-terminal intracellular segment which functions as a signal peptide for secretion and a stop-transfer signal for membrane anchoring, and a type II that has an extracellular N-terminal segment [6].

Because of its pathogenicity, virulence and high level of drug resistance, *Staphylococcus aureus* sortase A (SrtA) is the most extensively studied sortase and is regarded as a functional model for the development of inhibitors against Gram-positive bacteria [7]. SrtA is a type I sortase with 206 amino acids and has an eight-stranded β-barrel fold that includes two short helices and several loops [6]. The catalytic domain consists of His120, Cys184 and Arg197. The enzymatic mechanism proceeds via proton transfer from histidine-120 followed by the nucleophilic attack of the thiolate anion of the cysteine-184 residue [8]. The enzyme recognizes the target proteins by the C-terminal amino-acid sequence LPxTG and cleaves between the threonine and the glycine and joins the terminal amino group with the glycine residue of various substrates such as the peptidoglycan intermediate lipid II [9].

The discovery of the importance of the cysteine-184 residue and the inhibition of SrtA by various electrophilic thiol inactivators represented the starting point for the rational development of sortase inhibitors [10]. The development of efficient SrtA inhibitors employed by examining natural products, high-throughput screening of chemical libraries, or docking studies, but a clinical useful solution has not been discovered yet [11]. Chemically, a large diversity of classes can be described: vinyl sulfonyl derivatives [12], diarylacrylonitriles [13], aryl 3-acryloamides [14], aryl β-aminoethyl ketones [15], pyrazolethiones [16], rhodanines [5], pyridazinones [16], flavonoids [17], indole and bis(indole) alkaloids [18], benzisothiazolinones [19], triazolothiadiazole[20], and β-carboline derivatives [21].

The mechanism of SrtA inhibition has been studied for a few chemical classes, but it proved to be different depending on the chemical structure. Some SrtA inhibitors contain a α,β-unsaturated system capable to undergo a Michael addition with the thiol group from the cysteine-184 residue and to form a covalent adduct [22]. For some compounds, this structural feature is not present, but it can be generated by the enzyme in the catalytic process. In the case of 3-(dimethylamino)-1-(2-thienyl)-1-propanone, mass spectrometry and X-ray crystallography studies revealed that the inhibition mechanism is based on the elimination of the dimethylamino group and formation of the thienyl vinyl ketone which covalently binds to the cysteine’s thiol [15]. Methanethiosulfonates inhibit SrtA by forming a disulfide bond with the cysteine residue [23], whereas alkylating reagents such as N-ethylmaleimide, iodoacetate, and iodoacetamide proved to be inactive [24]. For a great number of compounds it have been concluded experimentally that the inhibition process is non-covalent, even if some of them contain reactive functionalities [25]. All this observation render very difficult a unified quantitative structure–activity analysis for all the SrtA inhibitors.

Several computational studies of the binding interactions of various ligands to SrtA revealed important structural features, but their utility is mostly restricted to a specific chemical scaffold. In this study we used a broader approach, trying to model simple parameters that can indicate potential new sortases inhibitors based on classification methods. Data mining methods can be used in the drug design process as a prerequisite sorting of candidates for further experimental tests based on structure derived descriptors, and molecular fingerprints [26]. We report the development of classification rules to predict low active or high active SrtA inhibition from molecular descriptors and chemical scaffolds.

## 2. Results

### 2.1. Data Description

A database (set 1) of 156 small molecules experimentally tested on SrtA was collected including 141 compounds having known IC_50_ values (set 1A), and 14 molecules with unknown IC_50_ values (set 1B) situated over a certain threshold. For the first group, the IC_50_ values ranges between 0.2 and 2680 µM, with an average value of 192.21 µM and a standard deviation of 416.45. A number of 15 structural descriptors were collected from PubChem and analyzed in order to understand the structural profile of the SrtA inhibitors.

All the compounds in the database are small organic molecules composed of carbon and hydrogen, and in some cases contain oxygen, nitrogen, sulfur or halogen atoms. There is no molecule that contains neither oxygen nor nitrogen. The average value of MW is 342.6 g/mol, the standard deviation is close to 94, and about 73 percent of the data values are within one standard deviation of the mean. The small average molecular weight could indicate a narrow binding site on the enzyme.

The analysis of the hydrogen bonding descriptors values indicates that all compounds have a HD value up to 5, with 94.3% in the range of 0 to 3. All the SrtA inhibitors in the set have at least one hydrogen acceptor. The average HA value is close to 4.6 and 93.6% of the compounds have a HA ranging from 2 to 6.

Rotatable bonds are defined as any single non-ring bond, bounded to nonterminal heavy atoms and their number represents a measure of molecular flexibility. The average RTB is close to 3.5 indicating the importance of a rigid molecular scaffold.

The number of compounds that contain a fluorine (F), chlorine (Cl), bromine (Br), iodine (I) was also computed. The fluorine can be found in 5.0% of the compounds, the chlorine atom in 14.9%, the bromine in 20.6%, and the iodine atom in only 0.7% of the compounds. Only 8.5% of the compounds have more than 2 halogen atoms in their structure.

The analysis of the number of rings revealed that all the compounds contain in their structure at least one ring, 91.49% of them containing between 2 and 5 cyclic structures. The benzene ring appears in 87.2% of the compounds.

A descriptive analysis of each set S, M and L was performed, in order to identify significant differences between the mean value and the range of variance of each structural descriptor. The data is presented in Table 1.

### 2.2. Bemis-Murcko Scaffold Analysis

Bemis and Murcko developed a simple method to identify structural scaffolds from two-dimensional molecular structures by removing the side chain atoms, and all atom labels. All bonds are set to single. These modifications generated so-called cyclic skeletons, frameworks with only the rings and the linker atoms connecting them, and account for the molecular topology [27].

**Figure 1:**
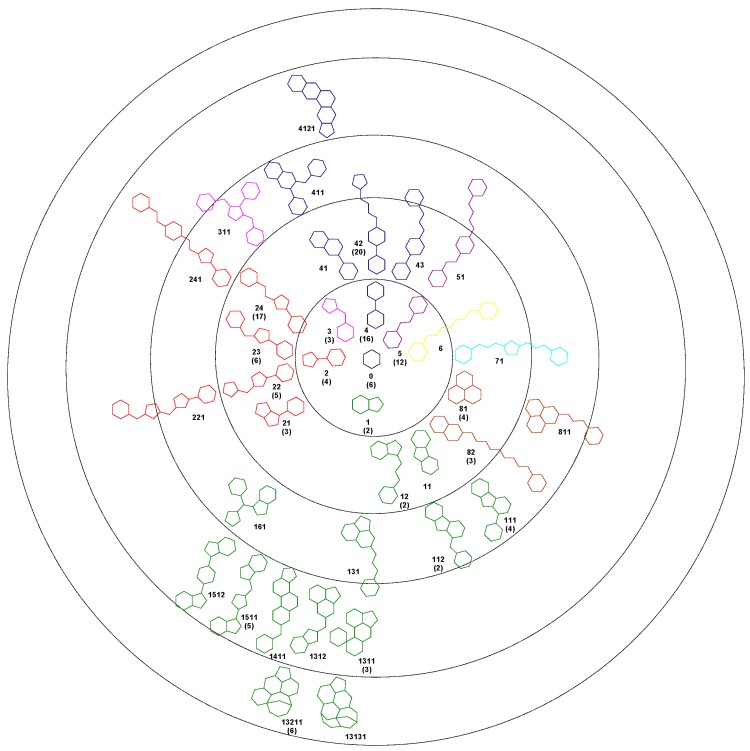
Bemis-Murcko scaffolds clustering for SrtA inhibitors, in brackets the number of compounds in the 1A set.

Using the Bemis-Murcko algorithm, we obtained 37 unique scaffolds and we hierarchical classified them based on the number of rings. Each scaffold was assigned to a node in the tree following an evolutionary method. With the exception of one compound, all the 140 structures in the set 1A can be described as deriving from a centroid hexagon. The first level of the cluster contains 6 scaffolds with two rings, the next level containing scaffolds with three rings, and so forth. For each scaffold a code number was assigned. The number of compounds based on that framework is presented in bracket. The colour of each scaffold represents the level 1 structure from which it derives (Figure 1).

The generated Bemis-Murcko scaffolds contain one to six pentagonal or hexagonal rings and have a number of atoms in the range of five to 27. Of the set of 37 scaffolds, a number of 16 appear in three or more compounds. For these scaffolds we performed a series of independent samples *t*-test analysis of the IC_50_ values, comparing the compounds which share the same scaffold with all the remaining compounds of the set 1A. For 12 scaffolds the average IC_50_ values are smaller than the left out compounds, indicating a favourable framework, but only for the scaffold 24 a significant difference was observed between the two groups (*p* < 0.05). In the case of scaffolds 2 and 21 the IC_50_ values are significantly higher than the rest of the set, indicating unfavourable chemical frameworks.

A similar leave-one-out procedure was performed using independent samples *t*-test analysis in order to understand if the number of rings in each scaffold is correlated with the IC_50_ value. For the compounds containing the scaffold 0, the average IC_50_ value is higher than the average of the rest of the compounds in the set. The same unfavourable effect on the SrtA inhibition appear to be produce by the scaffold containing two or three rings. Conversely, the subsets containing the four ring scaffolds, five and six rings scaffolds, have an average IC_50_ smaller than the left out compounds, but the differences are not statistical significant.

A linker value (Lnk) was defined as the number of bonds that connect two opposed rings in each scaffold and was computed following the shortest path. The linker values ranged from 0 to 12, with an average closed to 4.6. The analysis of the correlation between the linker and the IC_50_ values indicated that the best choice is a value between 4 and 7. In this case the IC_50_ average value is close to 74, in contrast with the rest of the compounds having an average IC_50_ close to 257, the difference being statistically significant (*p* < 0.05).

In order to understand the relevance of the side chains, we computed the number of atoms in each scaffold and for each compound we calculated the difference between MW and the weight of the carbon scaffold (DMW). This descriptor ranged from 18.1 to 494.6 with an average value of 135.3. When reported to the whole MW value, the DMW represented a percent varying 7.3 to 71.6, with a mean value of 38.9, pointing out the significance of bulk side chains.

### 2.3. Structural Cluster Analysis

The SrtA inhibitors of set 1A are classified using the PubChem single linkage clustering algorithm based on the structure similarity. The structure similarity is computed using the Tanimoto score calculated from the 2D structure fingerprint. PubChem uses an 881 substructure-keys (skeys) in each fingerprint, each bit representing the presence of a particular chemical group.

The smallest similarity score was 0.153 and was registered for the sitogluside and 4,5-dichloro-2-(3,5-dichlorophenyl)pyridazin-3-one pair, and the highest score was recorded for a pair of E/Z isomers. The compounds were assigned in 68 groups, of which 39 contain only one structure. This chemical diversity confirms the similar heterogeneity registered in the Bemis-Murcko scaffolding. The cluster with the highest compounds density contains 11 structures, followed by two clusters with seven elements. In the case of the 3D cluster using shape and feature resulted 55 groups. The two most populated groups contain 18 structures each.

### 2.4. Strong Inhibitors *Versus* Weak Inhibitors

An independent samples *t*-test was performed in order to determine whether there is a statistically significant difference between the mean values of any structural descriptors in the group containing strong inhibitors (S) and that of weak inhibitors (L). A number of 17 descriptors were analyzed, *p* value of 0.05 being considered statistically significant. In the case of MW, nC, nX, nS, HD, PSA, CLP, HVA, DMW and Lnk, no significant difference was recorded between the two groups, indicating that these structural characteristics are not important for the inhibitory potency. In Table 2 are presented the significant descriptors and the mean difference between the L and S groups.

**Figure 2:**
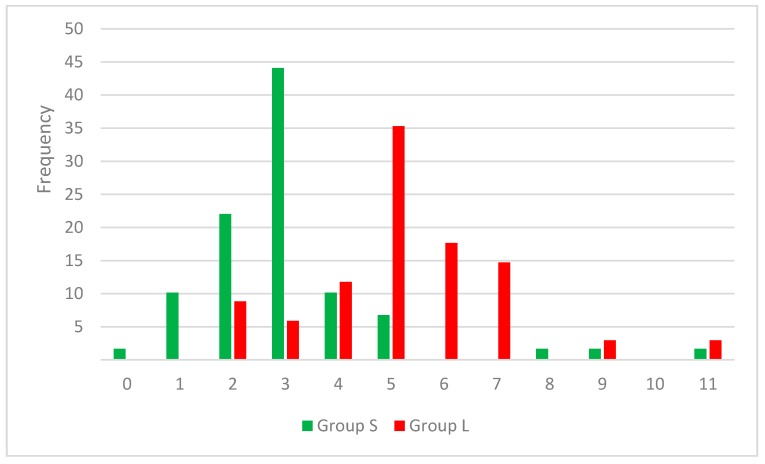
Combined histogram for RB frequency in strong (S) and low (L) inhibitors sets.

The results highlight the importance of a smaller number of rotatable bonds for a higher inhibitory effect on SrtA, translated in a more rigid framework. In the set S, 65% of the compounds contain two or three rotatable bonds, while in set L a proportion of 53 have a 5 or 6 RB value. In Figure 2 the distribution of RB values across both cluster of compounds can be observed.

**Figure 3:**
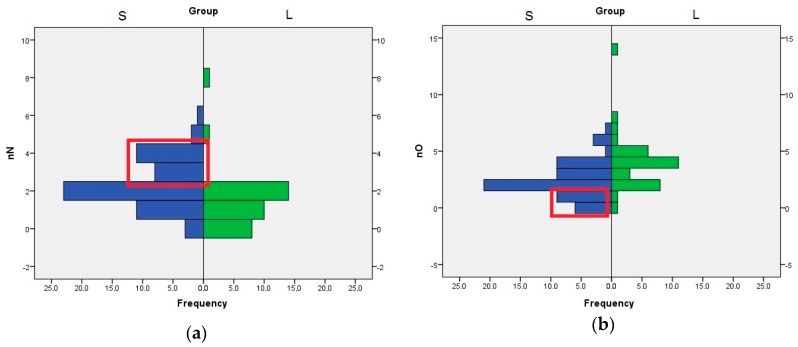
Histogram of nN and nO values across both sets of inhibitors, S and L (**a**) Distribution of nN values; (**b**) Distribution of nO values. The significant differences are highlighted in the red box.

The role of nN, nO, and HA descriptors are interconnected, because the HA value is computed by adding the number of nitrogen, oxygen, and fluorine atoms with free lone pairs of electrons. The distribution of nN values indicates that the compounds with three or four nitrogen atom are better SrtA inhibitors. In the case of nO, the compounds with one or none oxygen atom are found almost exclusively in the S set (Figure 3). Even if the means analysis indicated that a higher LogP is correlated with greater SrtA inhibitory potency, the comparison of the LogP histograms across both sets indicated that LogP values below 2 are a good predictor of low effect.

For all the descriptors presented in Table 2, demonstrated to have a statistical impact on the SrtA potency, a ROS curve analysis was performed to measure the performances of the classification method. The ROC curve is a graphical representation of the true positive ratio versus the false positive ratio. The best results were obtained for RB descriptor with an area under the curve (AUC) value of 0.839, and for nH descriptor where the AUC value was 0.794. The ROC curves were used to choose the best cut-off values, considering the higher importance of sensitivity over specificity in finding new chemical leads. A positive SrtA inhibitor can be considered if RB is less than equal to 4, test with a 0.88 sensitivity and 0.74 specificity. For nH, the cut-off was set to less than or equal to 18, the test having a 0.93 sensitivity, but only 0.44 specificity.

**Figure 4:**
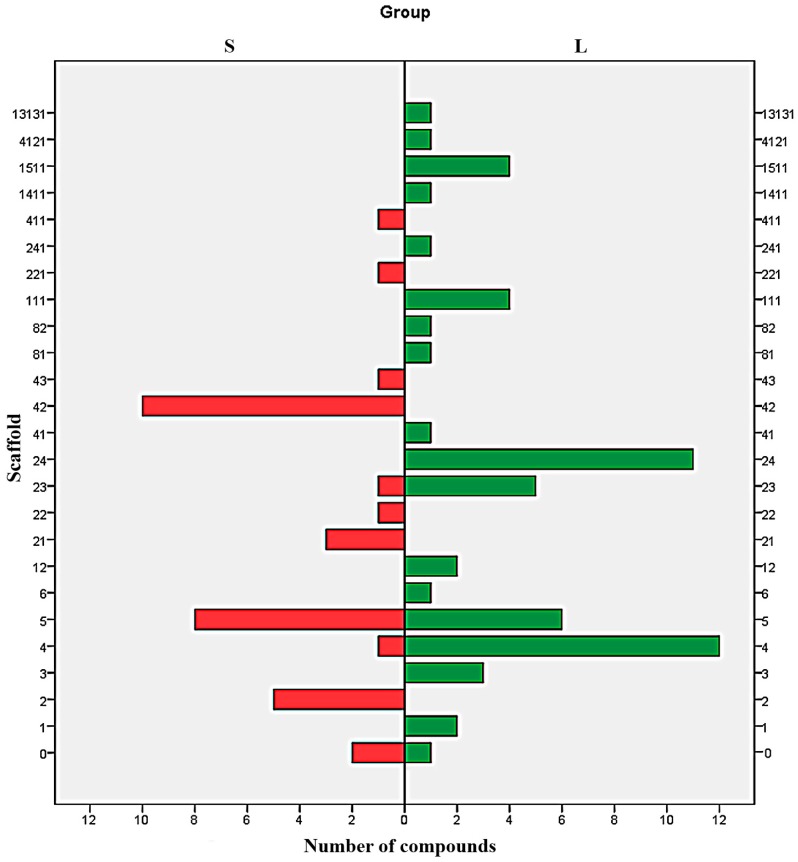
Parallel distribution of scaffolds in strong (S) and low (L) potency inhibitors.

Analysis of distribution of each type of scaffold in both S and L set shows a clear difference between the majorities of Bemis-Murcko structures (Figure 4). With the exception of the scaffolds 6, 0, and to some extent 4 and 23, all the scaffolds are exclusively distributed in just one subset. The results indicate the structures 2, 21, 22, 221, 42, 43 and 411 as undesirable and the scaffolds 1, 3, 12, 111, 13131, 141, 1511, 24, 241, 41, 4121, 81 and 82 as favourable. The scaffold 5 can be used to design potent SrtA inhibitors providing the proper addition of functional groups.

## 3. Discussion

The research was focused on a broad approach towards finding new sortases inhibitors, as possible anti-virulence drugs. Based on the PubChem data a number of 141 compounds with known IC_50_ data on SrtA, were identified and various analyses were performed in order to understand the correlation between the structure and the biological activity.

The structural set presented a good chemical diversity, even if some compounds shared a very high Tanimoto score, ensuring a large applicability. The results of the various analysis performed in this study are consistent, a clear portrait of a good SrtA inhibitor emerging as a small molecule with a MW between 180 and 600, containing one up to four nitrogen atoms, up to three oxygen atoms and under 18 hydrogen atoms. The number of oxygen and nitrogen atoms should be correlated so that the HA value must be greater than or equal to 2, but no higher than 7. Also, the HD value should be maximum 5. The candidates should have a low molecular flexibility with a RB value less or equal to 4, the RB value emerging as the most relevant descriptor for SrtA affinity.

The scope of this research was not to produce a complicated QSAR formula, often very hard to convert back to optimal chemical structures, but a simple tool for future explorations of the chemical space using structural descriptors as filters. Even if the provided rules of thumbs are relatively similar with other general drug-like and lead-like rules, they offer a comprehensible guideline for any future organic synthesis efforts.

In the prediction group (set 1), 99.4% of the compounds have a HA value equal or over 2, indicating the high importance of this descriptor, but for a potent inhibition, the HA value should not exceed 5. The results of our study are in concordance with the work performed by Uddin et al [28] which provided a pharmacophore model based on three hydrogen bond acceptor and two hydrophobic regions. The necessity for hydrophobic regions is confirmed by the requirement of LogP values above 2.

The Bemis-Murcko scaffolding revealed favoured scaffolds and scaffolds to should be avoided. All the preferred scaffolds contain at least two ring structures bond directly or merged in a condensed cycle. The drawback of this approach remains the limited variation of the side chains attached to the scaffolds. It is still possible that proper substitution on the basic framework to improve significantly the SrtA inhibitory effect. Despite its shortcomings, the characterization of the chemical architecture of SrtA inhibitors provides a straightforward method for designing new candidates, a major advantage over the QSAR methods.

These established data mining algorithms provide valuable a tool to search new potent SrtA inhibitors as potential anti-virulence agents targeted against *Staphylococcus aureus*, the reduction of the number of compounds used in enzymatic screens, and an enhancement of the screening yields.

## 4. Materials and Methods

PubChem is a public molecular repository established by the National Center for Biotechnology Information (NCBI), as a division of the National Institutes of Health (NIH). The PubChem directory is organized as three linked databases, PubChem Substance, PubChem Compound, and PubChem BioAssay [29]. The PubChem database (https://pubchem.ncbi.nlm.nih.gov/) was searched to find all the substances reported active on SrtA. The resulting compounds database was considered the training set and was analyzed in respect to the structural descriptors, molecular weight (MW), calculated logarithm of the octanol/water partition coefficient using the XLogP method (LogP), hydrogen bond donors count (HD), hydrogen bond acceptors count (HA), topological polar surface area (PSA), rotatable bonds count (RB), measure of structural complexity (CPL), degree of unsaturation (DOU) and carbon, hydrogen, oxygen, nitrogen, sulphur and halogen atom counts (nC, nH, nO, nN, nS and nX), heavy atoms number (HVA), existence of E/Z isomerism (EZ), and number of rings (NR). The half maximal inhibitory concentration (IC_50_) value was used as measure of the compounds effectiveness.

Based on the IC_50_ data, the database was divided in three subsets of SrtA inhibitors, a first set (S) containing strong inhibitors with IC_50_ values under 50 µM, the second set (M) of medium inhibitors with IC_50_ values between 50 and 250 µM and the third set (L) of low inhibitors, compounds with IC_50_ values over the 250 µM threshold. The cut-off values were selected based on pharmacological relevance and in order to ensure balanced dataset sizes. Independent samples *t*-test and ROC curves analyses were performed on the IBM Statistical Package for Social Sciences (SPSS) version 20 (Armonk, NY, USA).

## Figures and Tables

**Table 1:** Descriptive statistics for the structural descriptors of each SrtA inhibitors set.

Descriptor	Set S (59 Compounds)			Set M (63 Compounds)			Set L (34 Compounds)		
	Min	Max	Mean	Min	Max	Mean	Min	Max	Mean
IC_50_	0.2	48.0	18.5 ^a^	51.0	249.0	123.7 ^a^	255.3	2680	-
MW	183.3	576.8	324.1 ^a^	200.2	794.9	360.3 ^a^	168.2	640.5	335.3
nC	9	35	16.2	8	31	17.6	8	24	17.0
nH	7	60	14.6 ^b^	4	47	16.0	8	27	17.7 ^b^
nX	0	2	0.4	0	5	0.5 ^c^	0	2	0.2 ^c^
nN	0	6	2.4 ^b^	0	4	2.2 ^c^	0	8	1.5 ^b,c^
nO	0	7	2.5 ^a,b^	0	8	3.1 ^a,c^	0	14	4.0 ^b,c^
nS	0	2	0.6	0	3	0.7	0	2	0.7
DOU	4	17	10.9 ^b^	5	17	11.4 ^c^	5	16	9.8 ^b,c^
LogP	−2.0	7.7	3.2 ^b^	−0.1	7.5	2.9	−1.0	5.4	2.4 ^b^
HD	0	5	1.4	0	4	1.6 ^c^	0	4	0.9 ^c^
HA	1	8	4.2 ^ab^	2	11	4.8 ^a^	2	14	5.2 ^b^
RB	0	11	3.1 ^b^	0	11	3.6 ^c^	2	11	5.3 ^b,c^
PSA	20.3	190.0	79.2	32.7	241.0	89.5	18.5	238.0	84.1
CPL	159	955	477.6 ^a^	308	955	570.0 ^a,c^	189	1070	480.0 ^c^
HVA	12	41	22.3 ^a^	15	40	24.5 ^a^	11	40	23.6

^a,b,c^ mark the significant differences between the mean values at *p* < 0.05; ^a^ groups S and M, ^b^ groups S and L, ^c^ groups M and L.

**Table 2:** Significant structural descriptors for SrtA strong vs. weak inhibitors.

Descriptor	Sig. (2 Tailed)	Mean Difference	95% Confidence Interval	
			Lower	Upper
MW	0.545	−11.21	−47.83	25.41
nC	0.331	−0.86	−2.61	0.89
nH	0.038 *	−3.11	−6.03	−0.18
nX	0.095	0.18	−0.03	0.39
nN	0.004 *	0.89	0.29	1.49
nO	0.000 *	−1.53	−2.36	−0.70
nS	0.529	−0.09	−0.40	0.21
DOU	0.049 *	1.05	−0.06	2.16
LogP	0.008 *	0.83	0.22	1.45
HD	0.054	0.47	−0.01	0.95
HA	0.020 *	−0.96	−1.76	−0.16
RB	0.000 *	−2.18	−2.97	−1.39
PSA	0.570	−4.81	−21.59	11.96
CPL	0.945	−2.47	−72.84	67.90
HVA	0.267	−1.24	−3.44	0.96

(*) statistically significant as *p* < 0.05.
